# IP-score correlated to endogenous tumour antigen peptide processing: A candidate clinical response score algorithm of immune checkpoint inhibitors therapy in multiple cohorts

**DOI:** 10.3389/fimmu.2022.1085491

**Published:** 2023-01-09

**Authors:** Yutao Wang, Kexin Yan, Ye Guo, Yi Lu, Hao Su, Hongjun Li

**Affiliations:** ^1^ Department of Urology, Chinese Academy of Medical Sciences, Peking Union Medical College, Peking Union Medical College Hospital, Beijing, China; ^2^ Institute of Dermatology, Chinese Academy of Medical Sciences and Peking Union Medical College, Nanjing, China; ^3^ Department of Clinical Laboratory, Peking Union Medical College Hospital, Chinese Academy of Medical Science and Peking Union Medical College, Beijing, China

**Keywords:** IP score, ICI therapy clinical response, gene signature, single-cell analysis, endogenous tumour antigen peptide processing

## Abstract

The processing of endogenous tumour antigen peptides was essential for anti-tumour immunity in the tumour microenvironment. A high degree of Endogenous tumour antigen peptide processing has been demonstrated to improve the prognosis of carcinoma patients. However, there is insufficient evidence to prove its effect on the clinical response to immune checkpoint inhibitor therapy. To undertake a more in-depth analysis of the effects of the aforementioned genes on immunotherapy, we constructed a gene set evaluation score system relevant to tumour endogenous antigen peptide therapy using the GSVA approach. This rating mechanism is known as IP score (IPs). Immediately afterwards, we used the TCGA pan-cancer cohorts to conduct a comprehensive analysis of 6 genes in the IPs, and the analysis results showed that these six genes were related to the proportion of CD8^+^ T lymphocytes in a variety of solid tumours. As a prognostic protective factor for solid tumours, patients had better prognosis outcomes in the group with high expression levels of the above genes. We analysed the differential expression of six genes between immune checkpoint inhibitor treatment response and disease progression groups using several treatment cohorts. The results revealed that after treatment with PD-1 or CTLA4 inhibitors, the expression levels of the above six genes were comparatively high in the effective group, but the expression of the signature genes was dramatically downregulated in the ICI-insensitive groups. This indicates that the 6 genes are related to the clinical response to ICI treatment. Finally, we used the GSVA method to evaluate the above signatures, and the results showed that PDCD1, CTAL4, CD274 and LAG3 were significantly higher expressed in the IPs high-expression group; therefore, based on the processing of endogenous antigenic peptides in tumours, a predictive score of clinical response to immune checkpoint inhibitor therapy composed of 6 genes(PSMB8/PSMB9/PSMB10/PSME1/PSME2/IRF1) was constructed, and the role of each independent variable in the signature in the solid tumour microenvironment and the impact on ICI treatment were comprehensively analysed. This study provides a candidate evaluation score for predicting clinical response to immune checkpoint inhibitor therapy.

## Introduction

Malignant tumours claim the lives of millions of people every year, and cancer has come to symbolise a danger to people’s lives and health. The incidence of cancer and mortality will continue to rise significantly in the future due to population growth and increased awareness of life and health among people (1). Malignant tumours have a substantial impact on the safety of public life around the globe, and it has been estimated that by 2060, cancer may become the leading cause of death; thus, it is important to understand the mechanism of tumour genesis and coping measures (2).

Surgical treatment, adjuvant cytotoxic chemotherapy, radiotherapy, oncogene-targeted therapy, immunotherapy, and other methods are primarily used to treat tumours. Although these methods have proven successful for patients with advanced tumours, the techniques mentioned above and strategies cannot achieve satisfactory efficacy. Immunotherapy is employed primarily in patients with enormous tumour burdens and advanced malignancies. Immunotherapy is a significant invention that offers hope to cancer patients and has produced promising outcomes in a range of cancers (3, 4).

Allison and Honjo have contributed to the pathways of cytotoxic T lymphocyte-associated protein 4 and programmed death 1 (PD-1) (5). PD-1 and CTAL4 have become the novel immune checkpoint inhibitor approaches (6). Today, chimeric antigen T cells, adoptive cell therapy, and oncolytic viruses are revolutionising cancer treatment (7, 8). Despite the exciting efficacy of immune checkpoint inhibitor therapy, it still faces low clinical response rates and large side effects (9–11). The lack of T cells, various mechanisms preventing T cell migration and invasion, low tumour mutation burden, low PD-1/PDL1 expression, hyper angiogenesis, and high expression of vascular endothelial growth factor (VEGF) are some of the main factors of clinical treatment’s poor effectiveness (12–15). According to a study, the modulation of local chemokines or suppression of VEGF expression boosted T cell migration and infiltration in malignancies (16). Tumour cell antigen peptides are phagocytosed and integrated into human leukocyte antigen (HLA) class I molecules, processed by proteasomes (17). APCs ingest tumour antigens and migrate to lymphatic organs to elicit a subsequent immune response (18, 19). Subsequently, studies have shown a significant positive correlation between PSMB8 and CD8^+^ T lymphocytes (20, 21).

Our previous research examined the co-expression network linked with CD8^+^ T cells in urothelial cancer. The results revealed co-expressed genes included PSMB8, PSMB9, PSMB10, PSME1, PSME2, IRF1, TAP1, etc. The majority of these genes are concentrated in the presentation and processing of endogenous tumour antigen peptides (22), PSMB8, PSMB9, and PSMB10 are the core subunits of the immune proteasome, and PSME1 and PSME2 are the regulatory subunits. Since the level of T-lymphocyte infiltration is closely related to the clinical response to immune checkpoint inhibitor therapy, we hypothesise that the level of the above genes may affect the outcome of the clinical response to ICI therapy.

In this study, we first demonstrated the relationship among PSMB8, PSMB9, PSMB10, PSME1, PSME2, and IRF1 in the immune micro-environment and immune score through TCGA whole cancer cohorts, and further, we proved the correlation between PSMB8, PSMB9, PSMB10, PSME1, PSME2, IRF1, and immune cells. The mechanism of these genes and its impact on clinical characteristics in several immune checkpoint inhibitor cohorts were further analysed. Finally, we enriched the aforementioned gene sets using the GSVA scoring method and generated a new scoring method, the IP-score, that can predict the outcome of ICI treatment.

## Material and methodology

### Data collection

The Cancer Genome Atlas (TCGA) database yielded 33 different cancer database categories. The clinical data was also downloaded, including age, gender, and survival event. Meanwhile, the dataset for single-cell RNA sequencing (scRNA-seq) was downloaded from the Gene Expression Omnibus (GEO) database (https://www.ncbi.nlm.nih.gov/geo/). The bladder cancer (BLCA) scRNA-seq datasets were downloaded from BLCA-GSE145137 and PRAD-GSE137829. The GSE145140 (23) data set contained three samples: the single-cell RNA sequencing of chemotherapy-resistant muscle-invasive urothelial bladder cancer. GSE137829 (24) is the prostate single single-cell research which report luminal-neuroendocrine transdifferentiation prostate cancer samples.

### Immune therapy cohorts

Immunotherapy cohorts were acquired from the Immune Checkpoint Blockade Therapy Atlas (ICBatlas) (25). ICBatlas is a database that provides complete expression resources and functional analyses for ICB therapy patients. It also analyses the expression between the ICB groups of Response and Non-Response (R versus NR) and the Pre-treatment and On-treatment groups (Pre vs On). It is the first database of ICB treatment expression resources, and we intend to provide useful information and hints for ICB therapy-related clinical research.

ICBatlas encompasses 1515 samples dealt with by using PD-1/PD-L1, CTLA-4 inhibitors (1388 RNA-seq samples and 127 RNA-microarray samples) throughout 9 exclusive most cancers consisting of pores and skin cutaneous melanoma (SKCM), renal carcinoma (RCC), urothelial most cancers (UC), hepatocellular carcinoma (HCC), non-small lung most cancers (NSCLC), gastric most cancers (GC), head and neck squamous carcinoma (HNSCC), malignant pleural mesothelioma (MPM), and glioblastoma (GBM) from 25 datasets. The detailed cohort information was uploaded in **Supplementary Table 1**. Genomic, transcriptomic, and matched scientific statistics from sufferers with metastatic urothelial cancers dealt with an anti-PD-L1 agent (atezolizumab) are reachable beneath the Creative Commons three license and can be downloaded from http://research-pub.gene.com/IMvigor210CoreBiologies.

The GSE78220 immunotherapy cohort was acquired from the GEO website (26). The annotation platform was GPL11154. The cohort consists of 28 patients with melanoma. Complete response, partial response, stable disease, and progressing disease were immunotherapy responses.

### Immune micro-environment database

Using various techniques, we investigated the impact of PSMB8, PSMB9, PSMB10, PSME1, and PSME2 on immune cells and immunological microenvironments in distinct solid tumours. There is a multitude of available databases for evaluating TCGA tumours. TIMER is a valuable tool for the systematic examination of immune infiltrates in the majority of cancer types. This webserver model affords immune infiltrates’ abundances estimated through more than one immune deconvolution method and permits customers to generate top-notch figures dynamically to discover tumour immunological, medical and genomic points comprehensively. EPIC (27) is a tool to estimate the proportions of different cell types from bulk gene expression data. CIBERSORTx (28) is an analytical device from the Alizadeh Lab and Newman Lab to impute gene expression profiles and supply an estimation of the abundances of member telephone kinds in a combined phone population, the use of gene expression data.

### GSVA analysis

Gene set enrichment analysis (GSEA) is a computational method to determine the significance and consistency differences between two biological states of a predefined data set (29). The IP-score was conducted by GSVA analysis, the geneset contained PSMB8, PSMB9, PSMB10, PSME1, PSME2 and IRF1.

### Single cell sequencing analysis

The Anchors function of the “Seurat” R package was utilised to integrate BLCA-GSE145137 and PRAD-GSE137829. The scRNA-seq analysis was performed as part of the study (30). After scaling the data, PCA analysis was employed to decrease the dimension and then used the UMAP feature for the visualisation. The R package “InferCNV” (31) and “CopyKAT” (32) were applied for the identification of malignant cells. And the annotation of stromal cells and immune cells was based on the specific markers. The Dimplot, FeaturePlot, and VlnPlot were used to visualise the expression of PSMB8 further.

### TMB analysis

Tumour RNAseq data (level3) and corresponding clinical information were obtained from the Cancer Genome Atlas (TCGA) dataset (https://portal.gdc.com). Spearman’s correlation analysis describes the correlation between quantitative variables without normal distribution. And the genes with significant mutation differences in the high and low IP groups were calculated. A P-value of less than 0.05 was considered statistically significant.

### Prognostic analysis

To analyse the association between genes and overall survival in cancer patients, a Kaplan - Meier analysis was conducted to determine the overall survival (OS) of TCGA cohort patients. We aimed to demonstrate that these genes, as predictive protective genes, had an impact on overall survival.

## Results

### Flow chat

**Figure 1:**
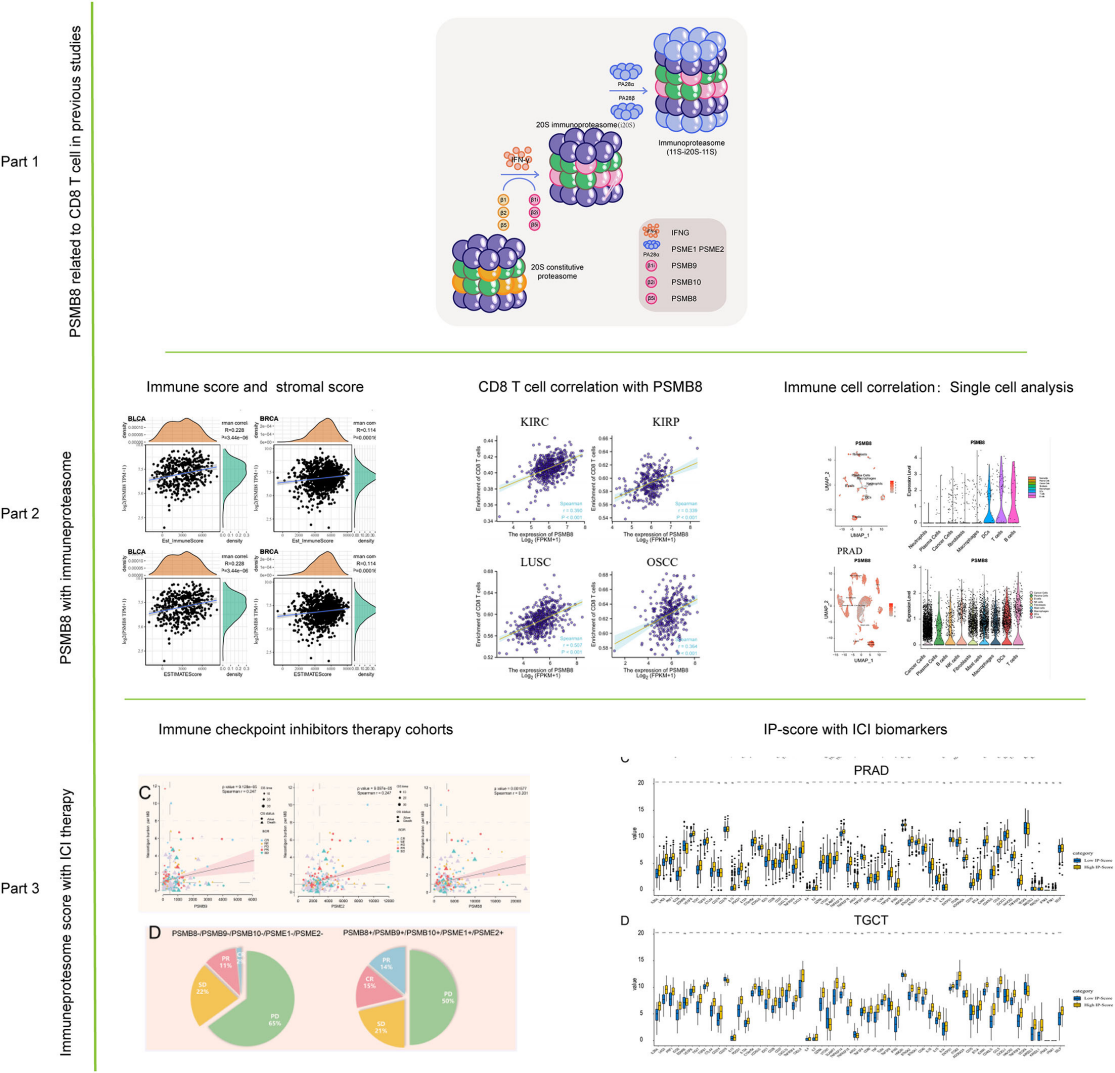
Flow chart of this study. The role of PSMB8 in cancer was obtained from previous studies. The correlation analysis of PSMB8 as an important gene of immune proteasome was studied by immune infiltration study and single cell analysis. Finally, the role of PSMB8 score in immunotherapy was studied.

Based on previous research, we discovered the CD8^+^ T lymphocyte-related co-expression network, in which PSMB8, PSMB9, PSMB10, PSME1 and other genes are primarily enriched in the functional subunits of immunoproteasome; therefore, we first presented the core subunits and regulatory subunits of immunoproteasome in Part 1 as well as images of the immunoproteasome-related mechanism in our previous article. In Part 2, we demonstrate the connection between PSMB8 and immune cells and the immunological microenvironment in additional solid tumours. In Part 3, we demonstrate that PSMB8 and IP-score can be utilised as biomarkers for ICI therapy and examine the relationship between IPS and immune checkpoint inhibitor medication (**Figure 1**).

### PSMB8 up-regulation correlates with immune response

The infiltration of immune cells into the tumour microenvironment has been shown to have a significant effect on the progression of cancer, but there is currently no effect of the genes mentioned above on the tumour microenvironment, so we carried out a thorough analysis of PSMB8 using the TCGA database. In our investigation, PSMB8 demonstrated a close association among different immune cells, including B cells and T cells, in KIRP, LGG, and LIHC (**Supplementary Figure 1A**).

LGG, TGCT, and THCA were the pan-cancer cohorts whose PSMB8 expression was most strongly correlated with the stromal score, and BLCA, BRCA, and CESC were the top three tumours whose PSMB8 expression was most strongly associated with the immune score. BLCA, BRCA, and LGG were the top three correlations between PSMB8 and ESTIMATE scores (**Supplementary Figure 1B**).

In addition, we mapped the connection between immune cell concentration and PSMB8 in TCGA other using the EPIC algorithm. The results demonstrate that PSMB8 was favourably and strongly linked with T cells and macrophages in various malignancies (**Supplementary Figure 1C**).

### The influence of the PSMB8 with immune checkpoint genes

Recent studies have found that immune checkpoint expression levels can be used as effective immunotherapy response biomarkers. Therefore, we summarised 47 immune checkpoint-related genes, and we discovered that PSMB8 is associated with immune checkpoints in a variety of solid tumours (33). We found that PSMB8 is related to immune checkpoints in a variety of solid tumours (**Supplementary Figure 1D**). In contrast, there was no significant relationship between PSMB8 and immune checkpoint in Lymphoid Neoplasm Large B-cell carcinoma. Similar results of PSMB9 and PSMB10 were uploaded as **Supplementary Figure 2** and **Supplementary Figure 3**.

### PSMB8 correlation with immune phenotype and clinical phenotype

**Figure 2:**
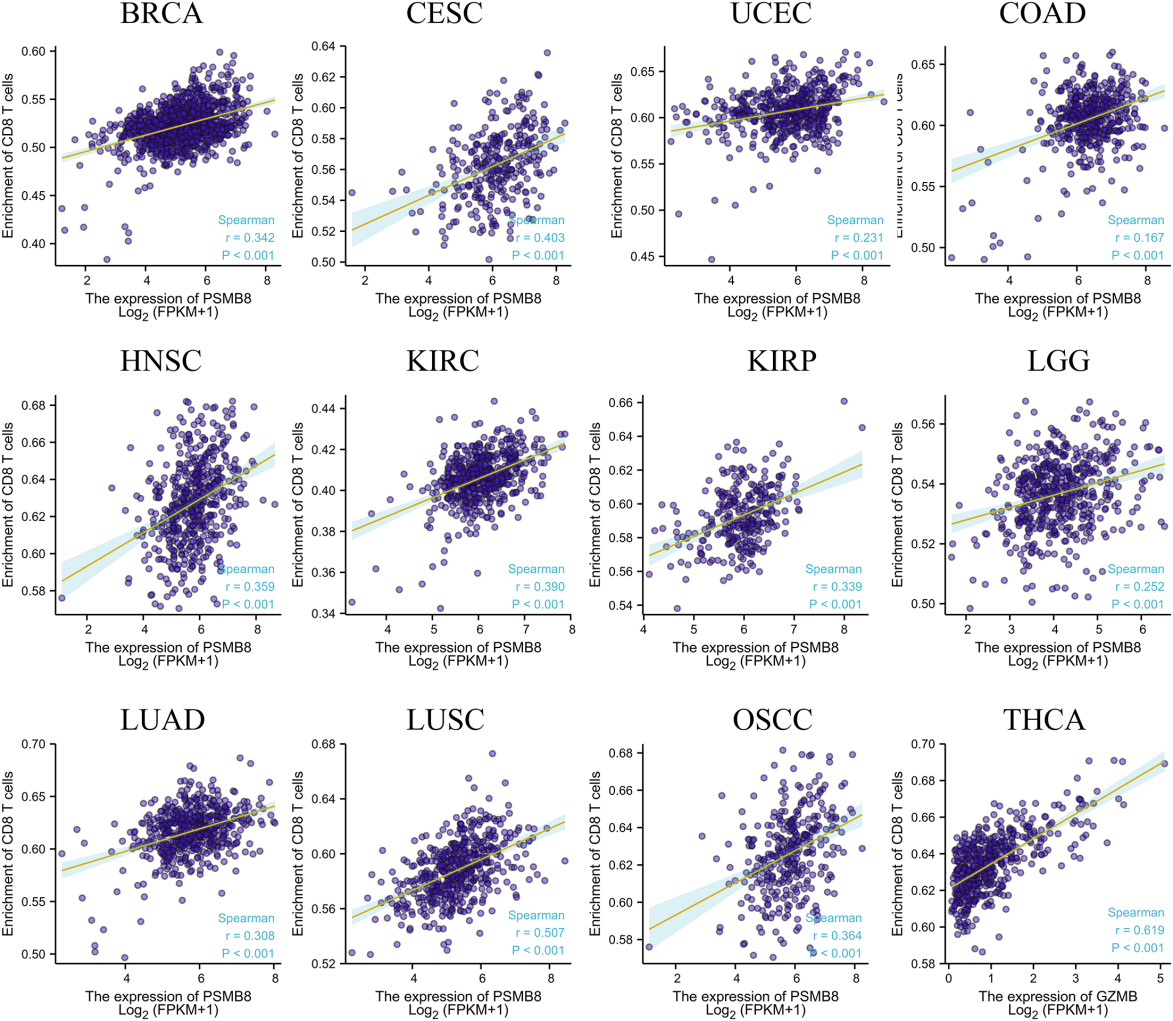
Correlation between PSMB8 expression and CD8^+^T cells in different cancers.

**Figure 3:**
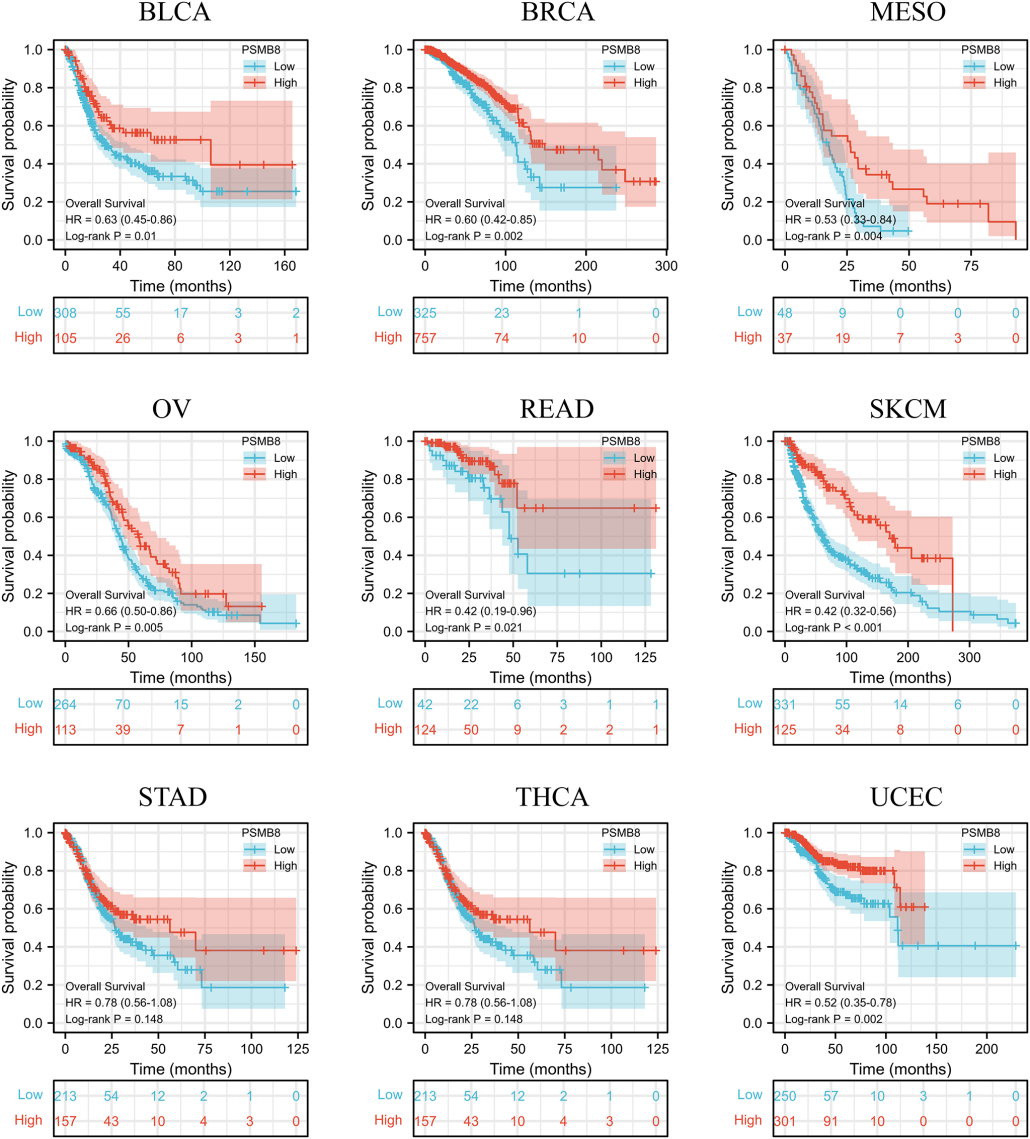
In different cancers, survival and prognosis analysis of PSMB8 showed that PSMB8 high expression group had a better prognosis.

Using the CIBERSORT method, we further evaluated the correlation between various T cell subtypes and PSMB8, and the results revealed a significant positive correlation between PSMB8 expression levels and CD8^+^ T cells in BRCA, CESC, UCEC, COAD, HNSC, KIRC, KIRP, LGG, LUAD, LUSC, OSCC, and THCA tumours (**Figure 2**). Moreover, The expression level of PSMB8 in KIRP, LIHC, LUAD, LUSC, OV, SKCM, SARC, PAAD, and PCPG tumours has significant positive correlation with macrophages (**Supplementary Figure 4**). Meanwhile, the overall survival of patients with the PSMB8 low expression group was worse in multiple cancers, including BLCA, BRCA, MESO, OV, READ, SKCM, STAD, THCA, and UCEC. These results suggest that PSMB8 may increase the anti-tumour response and further improve the prognosis of cancer patients (**Figure 3**).

### PSMB8 and T cell correlation based on single cell analysis

**Figure 4:**
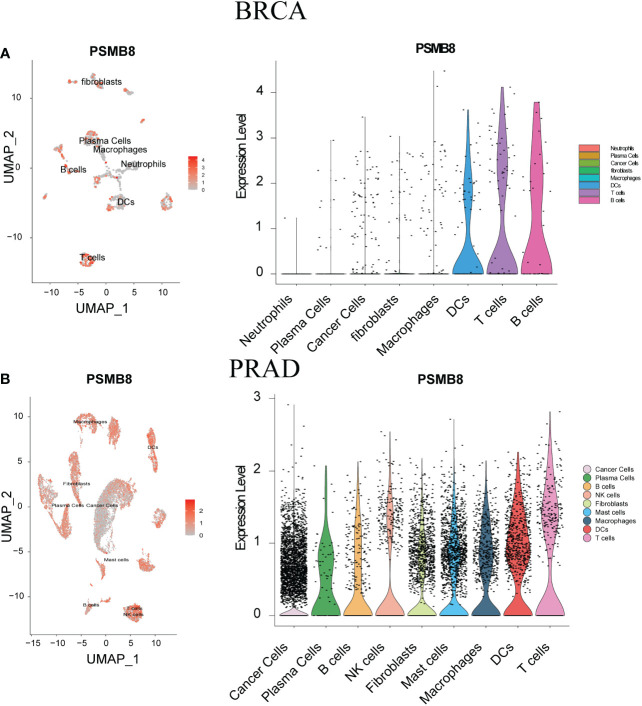
ScRNA-seq was used to show tumour cell localisation, cell classification and PSMB8 expression distribution. **(A)** The scRNA-seq results of PSMB8 expression in BLCA. **(B)** The scRNA-seq results of PSMB8 expression in PRAD.

We have analysed the distribution of PSMB8 and immune cells using two single-cell sequencing cohorts, BLCA - GSE145137 and PRAD - GSE137829. PSMB8 was shown to be substantially expressed on T cells in different malignancies. We determined the expression level and immune infiltration involvement of PSMB8 in BLCA and PRAD using scRNA-seq. In each tumour sample, distinct clusters of immune cell types were marked by immune cell-specific molecular markers. We discovered a significant association between T cells and PSMB8 (**Figure 4**).

### The PSMB8 acts as a biomarker for immune checkpoint therapy

**Figure 5:**
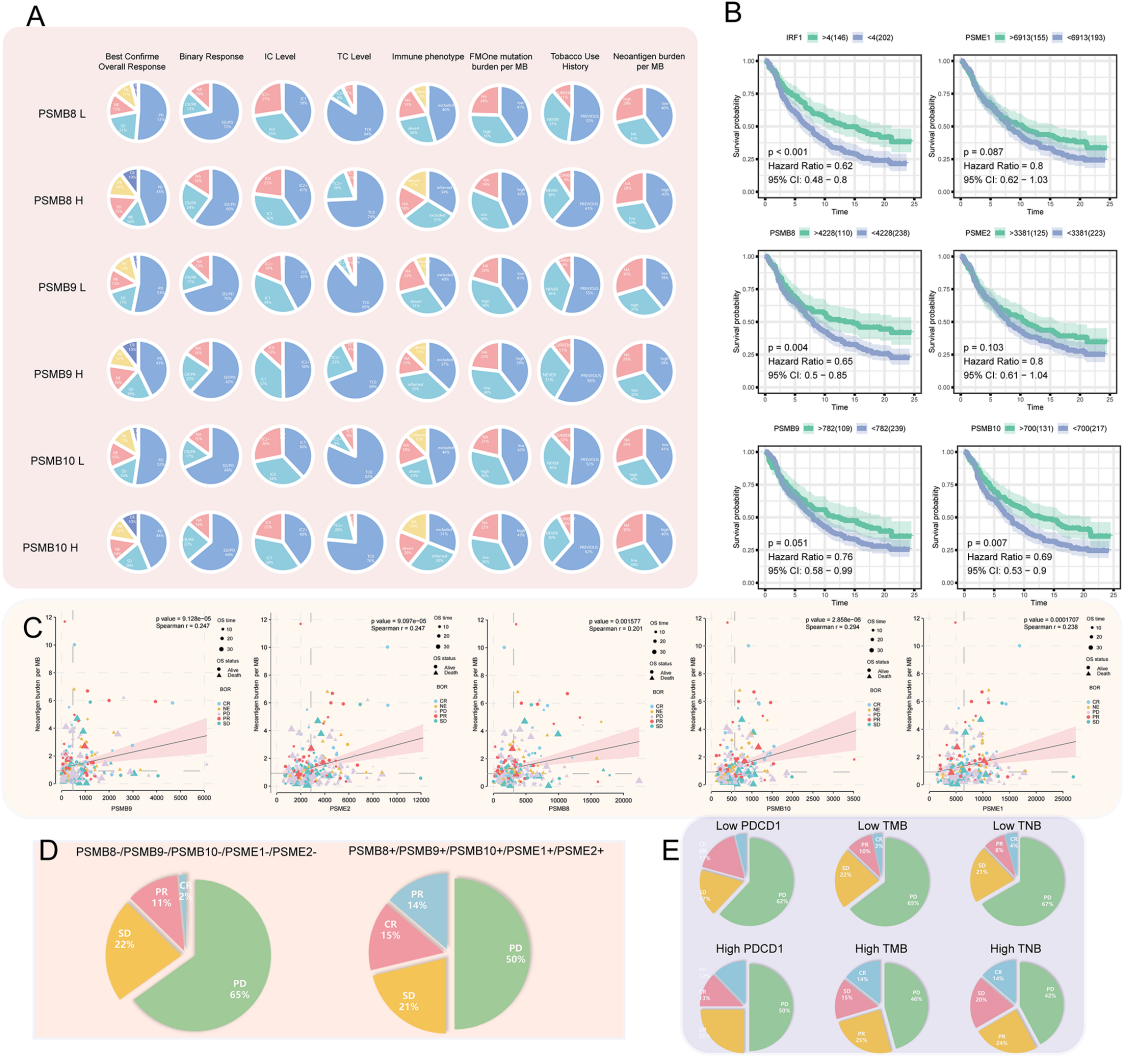
**(A)** Among the high- and low-expression groups of PSMB8, PSMB9, and PSMB10, difference analysis for the proportion of best confirmation of overall response, binary response, IC level, TC level, immune phenotype, FMOne mutation burden per MB, tobacco use history, and neoantigen burden per MB was carried out. In review CR, complete response; PR, partial response; NE, No effect; SD, Stable disease; PD, Progressive disease. **(B)** Survival analysis of IFNG (P < 0.001; HR = 0.62), PSME1 (P = 0.087; HR = 0.8), PSMB8 (P = 0.004; HR = 0.65), PSME2 (P = 0.103; HR = 0.8), PSMB9 (P = 0.051; HR = 0.76), and PSMB10 (P = 0.007; HR = 0.69) that are factors associated PSMB8. **(C)** In factors associated with the PSMB8, the correlation between CR, PR, NE, SD, and PD with neoantigen burden per MB. **(D)** In pan-cancer, the proportion of CR, PR, SD, and PD in the low- and high-expression groups of factors associated with the PSMB8. **(E)** The proportion of CR, PR, SD, and PD in the low- and high-expression groups of PDCD1 and TMB that have been reported as indicators of the efficacy of cancer treatment. Compared with Figure D, factors associated with PSMB8 had the strongest predictive ability.

**Figure 6:**
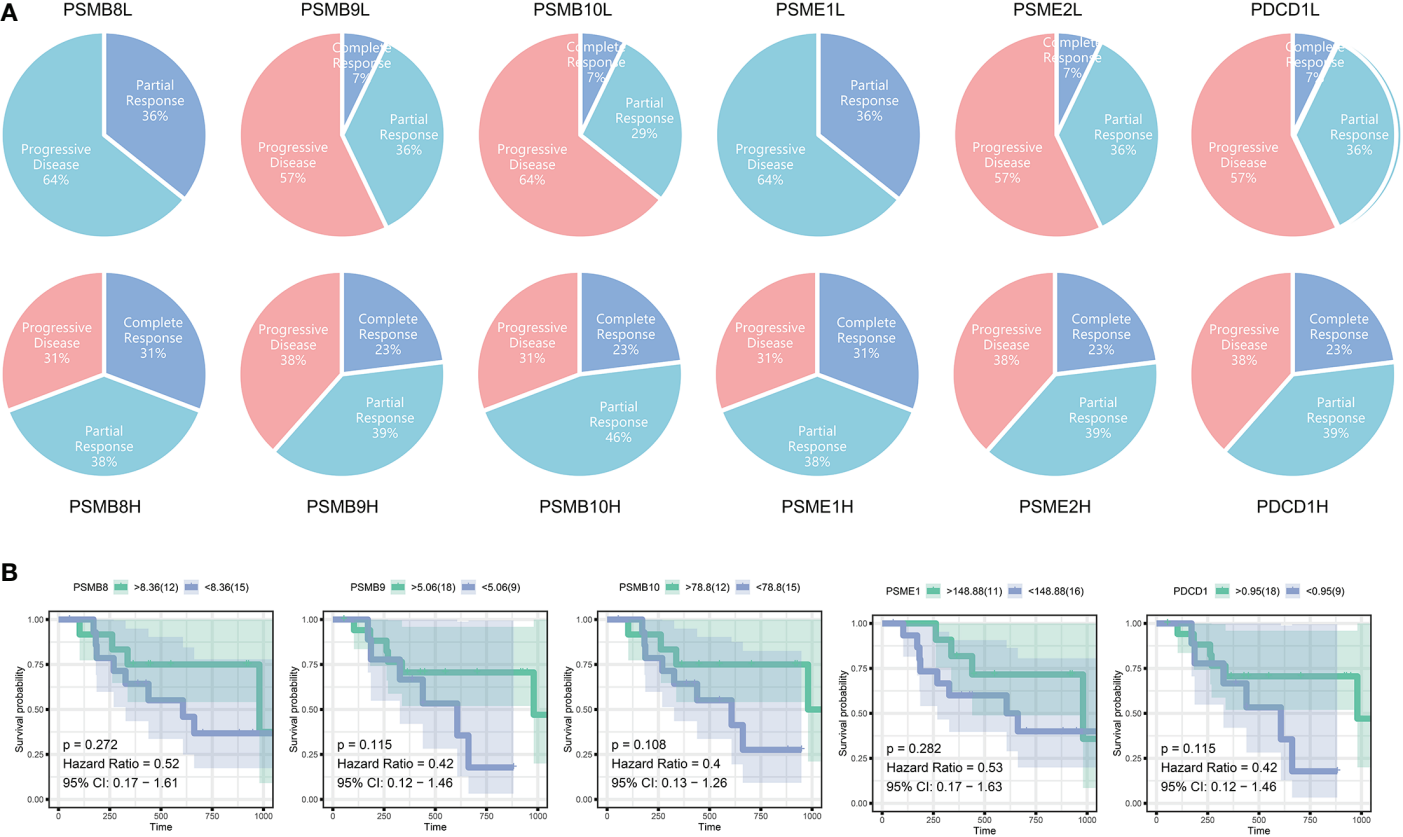
**(A)** The immunotherapy outcomes in GSE78220. **(B)** Survival analysis of different subunit genes of immune proteasome in cutaneous malignant melanoma.

Immunotherapy has been successful, although most people are not immunosensitive. In the tumour microenvironment, these insensitive patients had decreased levels of PD-1 expression and cytotoxic T cells. We believe that the PSMB8 concentration influences the CD8^+^ T lymphocyte concentration in the microenvironment. This effect may mitigate the immune checkpoint treatment’s insensitivity induced by the low cytotoxic T cell concentration. The immunotherapy cohort from IMvigor210 CoreBiologies was utilised. The cohort included 348 immune checkpoint treatment participants. The rates of disease progression in patients with high expression levels of the core subunits after using immune checkpoint inhibitors were lower than the patients with low expression levels. The PD-1 expression scores of tumour cells and immune cells in patients with high expression levels of PSMB8, PSMB9, and PSMB10 were greater than low expression samples (**Figure 5A**). Based on the greatest sensitivity and specificity of PSMB10, PSMB9, PSMB8, PSME1, PSME2, and IRF1, we then separated samples into two groups. We discovered that the overall survival rates of patients with low PSMB8 and IRF1 expression were higher. Other factors’ results were not statistically significant, although their trends were comparable to those of prognosis protective factors (**Figure 5B**). **Figure 5C** illustrates the connection between PSMB8 subunits and tumour mutation burden over a spectrum of immunotherapy response phases. Patients with complete responses had a greater positive connection between PSMB8 and tumour mutation burden. In conclusion, we discovered that PSMB8 improves outcomes and boosts immunotherapy’s complete response. For PSMB8^+^/PSMB9^+^/PSMB10^+^/PSME1^+^/PSME2^+^ (these genes are highly expressed) samples, the complete response rate of immune checkpoint treatment was 15%, and the partial response rate was 14%. For PSMB8-/PSMB9-/PSMB10-/PSME1-/PSME2- (these genes are lowly expressed) samples, the complete response rate of checkpoint treatment was 2%, and the partial response rate was 11% (**Figure 5D**). When the PD-1 expression level was used as the criterion for the efficacy of immunotherapy, 13% of patients in the PD-1 overexpression group had a complete response, and 12% had a partial response. In the PD-1 low-expression group, 4% of patients had a complete response, and 17% had a partial response. Using the tumour mutation burden level as the criterion for the efficacy of immunotherapy, 14% of patients had a complete response, and 15% had a partial response in the mutation burden overexpression group. In the mutation load low-expression group, 3% of patients had a complete response, and 10% had a partial response (**Figure 5E**). Finally, univariate Cox regression analysis was performed for all variables in the urothelial epithelial carcinoma immune checkpoint cohort, taking the time corresponding to the complete response as termination time. The same result was found in the GSE78220 melanoma cohort (**Figure 6**).

### IP - score correlation with immune inflammation response

**Figure 7:**
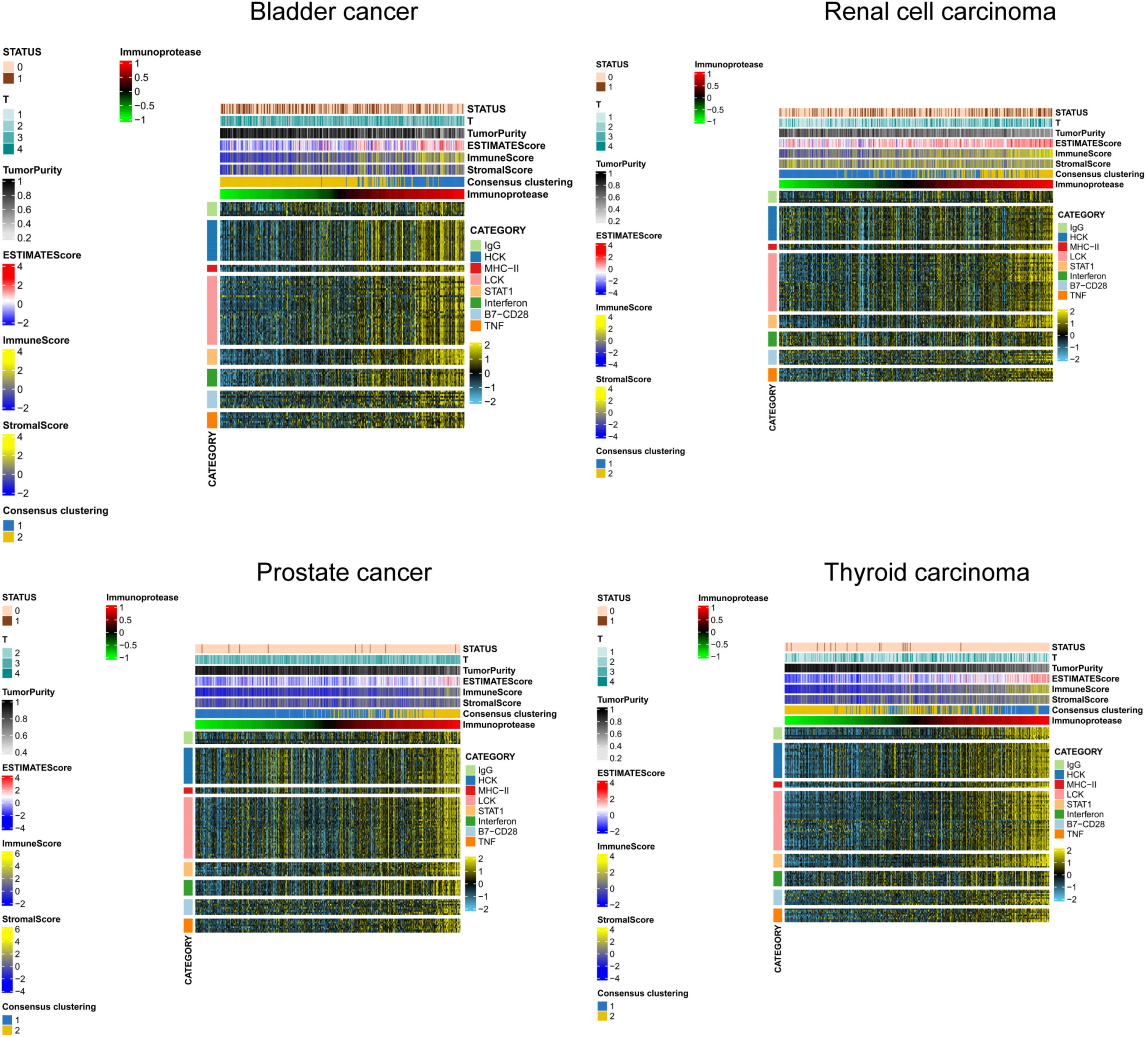
IP - Score correlation with immune inflammation response.

We selected several classic immune - related subgene sets, including major histocompatibility complex class II (MHC-II), lymphocyte-specific kinase (LCK), hematopoietic cell kinase (HCK), immunoglobulin G (IgG), signal transduction and activation transcription 1 (STAT1), co-stimulatory molecules (B7-CD28), interferon and TNF gene sets. We analysed the relationship between IP scores and immune inflammatory responses. We found that as the IP score increased, the igG immunoglobulin secreted by B cells increased, and the expression of biomarkers in macrophages and monocytes/myeloid cells also increased significantly. The content of histocompatibility class II complex, histocompatibility class I complex increased, and the expression level of surface markers of T cells and macrophages also increased significantly in KIRC, BLCA, PRAD and THCA (**Figure 7**).

### IP - score establishment and its role in TMB and immune check point genes

**Figure 8:**
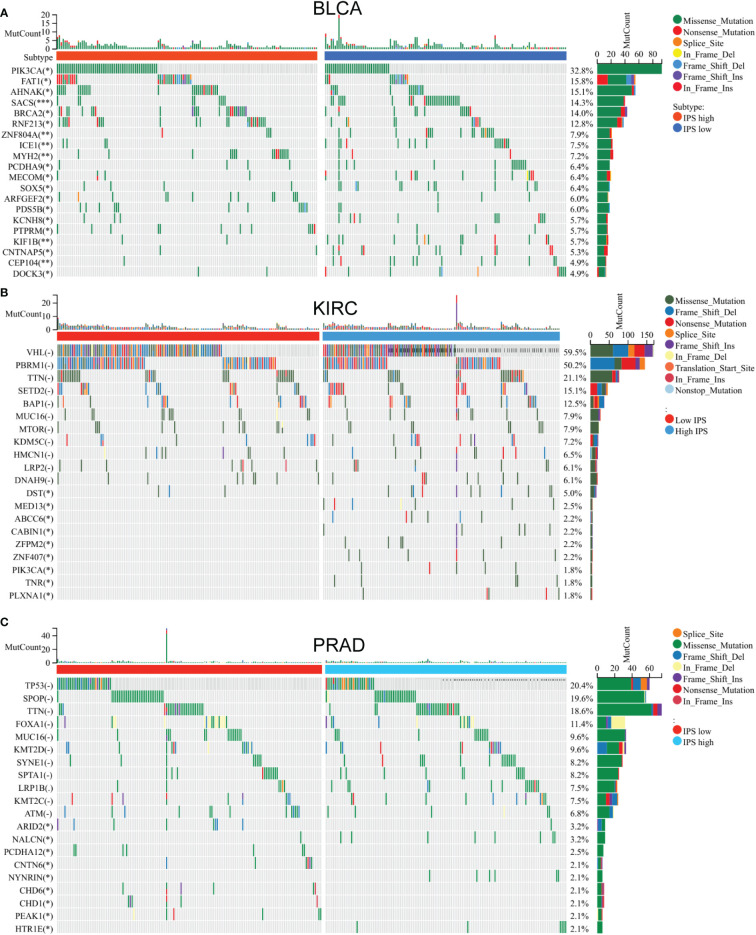
Mutation gene difference between low IP - score and high IP - score. Mutation gene difference between low IP - score and high IP - score in **(A)** Bladder carcinoma. **(B)** Kidney renal clear cell carcinoma. **(C)** Testicular Germ Cell Tumors.

Establishment of IP score and its function in TMB and immune checkpoint genes. We use the immune checkpoint database for PSMB8/PSMB9/PSMB10/PSME1/PSME2/IRF1 in light of the study above. Based on the above research, we use the immune checkpoint database for PSMB8/PSMB9/PSMB10/PSME1/PSME2/IRF1. The role of these genes in the immunotherapy queue was analysed, and the results show that PSMB8/PSMB9/PSMB10/PSME1/PSME2/IRF1 in multiple clinical response immune checkpoint inhibitor treatment group had expressed, in the stable group of disease, the low expression of queue these solid tumours including lung cancer, kidney cancer, bladder cancer, detailed gap analysis statistical data as shown in **Tables 1**–**5**. These results suggested that all six genes could be prospective biomarkers for predicting clinical response to ICI therapy. Then, using the GSVA approach, we assigned an IP-score to the gene set comprised of the above six genes. To verify the influence of IP-score on tumour mutation and immune response in greater detail, we initially examined the association between IP-score and mutation in solid urological tumours. The results demonstrated that the mutation frequencies of the PIK3CA, BRCA2, RNF213, and SACS genes in the urothelial cancer cohort were significantly different between low and high IP-score groups (**Figure 8A**).

**Table 1:** PSMB8 expression distribution for response and non-response based on pre-treatment samples in all datasets.

PSMB8	Study	Cancers	Anti Target	Response Mean	Non-Response Mean	Log2FC	FDR	P Value
1	ERP105482,SRP150548,SRP128156	Melanoma,RCC	anti-PD1 + anti-CTLA4	3,743.00	1,698.00	1.42	0.002	0.001
2	ERP105482	Melanoma	anti-PD1 + anti-CTLA4	4,685.00	1,698.00	1.519	0.003	0.001
3	ERP107734	Gastric Cancer	anti-PD1	6,223.50	3,111.00	0.862	0.01	0.001
4	ERP105482,SRP011540,SRP070710,SRP094781,SRP150548,SRP230414,SRP250849,SRP302761	Melanoma	anti-PD1/anti-CTLA4/anti-PD1 + anti-CTLA4	2,819.00	2,495.00	0.258	0.103	0.016
5	anti-PD1	Melanoma,NSCLC,GBM,RCC,GC	anti-PD1	2,466.50	2,410.00	0.215	0.121	0.024
6	ERP105482	Melanoma	anti-PD1	2,625.00	1,734.50	0.648	0.143	0.009
7	IMvigor210	Urothelial Cancer	anti-PDL1	4,453.91	3,642.02	0.253	0.262	0.089
8	SRP183455,SRP217040	NSCLC	anti-PD1/PDL1	2,434.00	1,652.00	0.577	0.299	0.064

**Table 2:** PSMB9 expression distribution for response and non-response based on pre-treatment samples in all datasets.

PSMB9	Study	Cancers	Anti Target	Response Mean	Non-Response Mean	Log2FC	FDR	P Value
1	ERP107734	Gastric Cancer	anti-PD1	5,739.00	1,407.00	1.303	0.002	0.001
2	anti-PD1	Melanoma,NSCLC,GBM,RCC,GC	anti-PD1	895	946	0.416	0.011	0.001
3	ERP105482,SRP150548,SRP128156	Melanoma,RCC	anti-PD1 + anti-CTLA4	1,253.00	305	1.466	0.028	0.001
4	ERP105482	Melanoma	anti-PD1 + anti-CTLA4	1,026.00	305	1.559	0.035	0.001
5	IMvigor210	Urothelial Cancer	anti-PDL1	918.88	674.66	0.418	0.075	0.012
6	SRP183455,SRP217040	NSCLC	anti-PD1/PDL1	1,368.00	711	1.035	0.078	0.006
7	ERP105482,SRP011540,SRP070710,SRP094781,SRP150548,SRP230414,SRP250849,SRP302761	Melanoma	anti-PD1/anti-CTLA4/anti-PD1 + anti-CTLA4	875	859.5	0.347	0.087	0.013
8	ERP105482	Melanoma	anti-PD1	711	262.5	0.865	0.134	0.008
9	GSE136961	Non-small Cell Lung Cancer	anti-PD1	13.1	11.63	1.462	0.169	0.012
10	SRP217040	Non-small Cell Lung Cancer	anti-PDL1	1,982.50	1,099.00	1.106	0.243	0.031

**Table 3:** PSMB10 expression distribution for response and non-response based on pre-treatment samples in all datasets.

PSMB10	Study	Cancers	Anti Target	Response Mean	Non-Response Mean	Log2FC	FDR	P Value
1	anti-PD1	Melanoma,NSCLC,GBM,RCC,GC	anti-PD1	384.5	379.5	0.486	0	0.001
2	ERP105482,SRP011540,SRP070710,SRP094781,SRP150548,SRP230414,SRP250849,SRP302761	Melanoma	anti-PD1/anti-CTLA4/anti-PD1 + anti-CTLA4	315.5	334.5	0.438	0.003	0.001
3	ERP107734	Gastric Cancer	anti-PD1	1,588.00	780	0.976	0.02	0.001
4	IMvigor210	Urothelial Cancer	anti-PDL1	807.81	639.4	0.281	0.031	0.003
5	ERP105482	Melanoma	anti-PD1	236	131	0.748	0.04	0.001
6	ERP105482,SRP150548,SRP128156	Melanoma,RCC	anti-PD1 + anti-CTLA4	374	178	0.964	0.073	0.001
7	ERP105482	Melanoma	anti-PD1 + anti-CTLA4	342	178	1.037	0.093	0.002
8	SRP183455,SRP217040	NSCLC	anti-PD1/PDL1	463	223	0.783	0.123	0.013
9	SRP011540	Melanoma	anti-PD1	268	267	0.411	0.211	0.033
10	SRP183455	Non-small Cell Lung Cancer	anti-PD1	198	142.5	0.767	0.235	0.029

**Table 4:** PSME1 expression distribution for response and non-response based on pre-treatment samples in all datasets.

PSME1	Study	Cancers	Anti Target	Response Mean	Non-Response Mean	Log2FC	FDR	P Value
1	ERP105482,SRP011540,SRP070710,SRP094781,SRP150548,SRP230414,SRP250849,SRP302761	Melanoma	anti-PD1/anti-CTLA4/anti-PD1 + anti-CTLA4	5,179.00	4,281.50	0.31	0.004	0.001
2	anti-PD1	Melanoma,NSCLC,GBM,RCC,GC	anti-PD1	4,481.50	4,047.00	0.25	0.008	0.001
3	ERP105482,SRP150548,SRP128156	Melanoma,RCC	anti-PD1 + anti-CTLA4	6,757.00	3,799.00	0.99	0.033	0.001
4	ERP105482	Melanoma	anti-PD1 + anti-CTLA4	6,825.00	3,799.00	1.105	0.04	0.001
5	ERP107734	Gastric Cancer	anti-PD1	6,480.50	4,259.00	0.437	0.115	0.013
6	ERP105482	Melanoma	anti-PD1	5,751.00	3,690.00	0.495	0.222	0.021
7	IMvigor210	Urothelial Cancer	anti-PDL1	7,837.81	7,005.39	0.12	0.228	0.071
8	SRP183455,SRP217040	NSCLC	anti-PD1/PDL1	4,281.50	3,004.00	0.472	0.352	0.09

**Table 5:** PSME2 expression distribution for response and non-response based on pre-treatment samples in all datasets.

PSME2	Study	Cancers	Anti Target	Response Mean	Non-Response Mean	Log2FC	FDR	P Value
1	ERP107734	Gastric Cancer	anti-PD1	7,174.50	3,535.00	0.773	0.015	0.001
2	anti-PD1	Melanoma,NSCLC,GBM,RCC,GC	anti-PD1	3,336.50	3,237.50	0.246	0.016	0.001
3	ERP105482,SRP011540,SRP070710,SRP094781,SRP150548,SRP230414,SRP250849,SRP302761	Melanoma	anti-PD1/anti-CTLA4/anti-PD1 + anti-CTLA4	3,918.00	3,710.00	0.244	0.033	0.003
4	ERP105482,SRP150548,SRP128156	Melanoma,RCC	anti-PD1 + anti-CTLA4	5,418.00	3,897.00	0.962	0.038	0.001
5	ERP105482	Melanoma	anti-PD1 + anti-CTLA4	5,905.00	4,668.00	0.937	0.108	0.002
6	IMvigor210	Urothelial Cancer	anti-PDL1	3,586.49	3,105.91	0.169	0.143	0.034
7	ERP105482	Melanoma	anti-PD1	4,174.00	2,862.00	0.546	0.156	0.011
8	SRP183455,SRP217040	NSCLC	anti-PD1/PDL1	3,612.50	1,957.00	0.477	0.325	0.075

**Figure 9:**
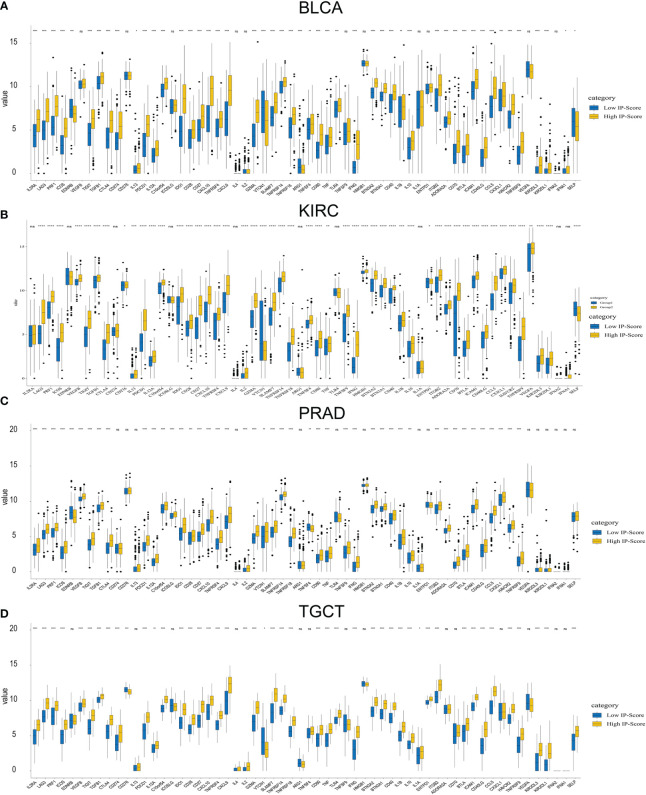
Immune check point expression difference between low ip-score and high ip-score. Immune check point expression difference between low ip-score and high ip-score. **(A)** Bladder carcinoma **(B)** Kidney renal clear cell carcinoma **(C)** Prostate carcinoma **(D)** Testicular Germ Cell Tumors. * represent p<0.05; ** represent p<0.01; *** represent p<0.001; **** represent p<0.0001; ns, represent non significant.

In the clear cell renal cell carcinoma cohort, the DST, MED13 and ABCC6 gene mutation frequencies significantly differed among different low and high IP-score groups (**Figure 8B**). In the prostate cancer cohort, the mutation frequencies of ARID2, CNTN6, CHD1 and other genes were significantly different in different IP-score groups (**Figure 8C**). In urinary solid tumours, the immune checkpoint genes such as LAG3/CTLA4/PD-1/PD-L1 were over-expressed in the group with high IP-score, while the immune checkpoint genes such as LAG3/CTLA4/PD-1/PD-L1 were low-expressed in the group with low IP-score. This indicates that the IP score is an essential predictor of immune checkpoint inhibitor therapy (**Figures 9A–D**).

## Discussion

Immune checkpoints can regulate the body’s immune system, among which stimulatory checkpoint molecules can promote the activation of T cells and activate the body’s immune response; As the immune system’s natural “brake,” inhibitory checkpoint molecules are utilised to restrict the body’s immunological response and avoid autoimmunity (34).

To avoid being eliminated by the body’s immune system, tumour cells suppress the body’s immunological response by producing inhibitory immune checkpoint molecules that interact with T cells (35–37).

Consequently, appropriate antibody medicines can be designed for common suppressive immune checkpoints, the body’s immune system can be boosted by blocking suppressive immune checkpoints, and the tumour is subsequently eliminated. More than a dozen immunological checkpoints have been identified, with CTLA-4 and PD-1/PDL1 being the most extensively investigated. CTLA-4 is a T cell surface receptor that transmits immunosuppressive signals and functions as an immunosuppressive molecule (34).

Research indicated that CTLA-4 deletion in mice models can result in enormous lymphocyte proliferation, organ damage, and even mouse death. Further research discovered that inhibiting CTLA-4 could significantly suppress tumour growth in tumour model mice (38). Following multiple clinical trials, the FDA authorised ipilimumab, the first antibody medication against the immunological checkpoint CTLA-4, in 2011 (39).

The antibody drug is mainly used for the treatment of melanoma, which can improve the survival of patients for 1~2 year. In addition to CTLA-4, PD-1 is a prominent immunosuppressive protein on the surface of T cells, and its ligand. PD-L1 is expressed in a variety of tumour cells. P. By binding to PD-1 on the surface of T cells, T cell activation is suppressed, resulting in tumour immune evasion. Inhibitors of PD-1/PD-L1 suppress these immunological checkpoints, increasing T cells’ activity and destroying tumour cells. In 2014, the FDA approved the first PD-1 inhibitor, pembrolizumab, for treating melanoma and lung cancer. In 2016, the FDA approved the first PD-L1 inhibitor, atezolizumab, to treat bladder cancer (40, 41).

Immune checkpoint blocking therapy for CTLA4 or PD-1 (PD-L1) has made great breakthroughs in the treatment of different types of tumours. However, only a subset of patients benefit. Therefore, analytical immunotherapy drivers of resistance and finding predictors are critical. The efficacy of immune checkpoint inhibitors is influenced by a variety of factors, including tumour genomics, host genetics, PD-L1 levels, tumour micro-environment and intestinal microbiome, etc (42). In addition, the use of new technology to analyse the heterogeneity of immune cells in the tumour micro-environment has important guiding significance for developing targeted therapies for immune cells and predicting the effect of immune checkpoint blockade therapy. The development of new technologies represented by single-cell sequencing has extensively promoted research in this field. Zhang Zemin’s research group (43) of Peking University used Smart sequencing technology (Smart-seq) to map the immune map of T cells at the single-cell level in lung cancer and colon cancer and comprehensively analysed tumour leaching. The subpopulation characteristics, cell heterogeneity, tissue distribution, and T cells’ drug target gene expression demonstrated the T cells’ dynamic alterations in the tumour microenvironment.

Our previous studies found a co-expression network associated with CD8^+^ T lymphocyte invasion in urothelial carcinoma, which contains PSMB8, PSMB9, PSMB10, PSME2, IRF1 and other genes. These co-expressed genes are mainly enriched in the processing and presentation of endogenous tumour antigen peptides. Further review of the literature found that these genes are mainly involved in the composition of the core subunits of PSMB8. PSMB8 have been extensively studied in tumours. However, their roles in the solid tumour microenvironment and its relationship to immune checkpoint inhibitor therapy have not been fully studied. Therefore, the TCGA database was initially utilised to investigate the impact of PSMB8 core subunit and regulatory subunit constituent genes on the tumour microenvironment of solid tumours and whether they may be utilised as predictors of clinical survival. Then, using several cohorts receiving PD-1/PDL1 and CTLA-4 treatment, we analysed the variations in the expression levels of the genes mentioned above between the responsive and insensitive groups of drug treatment to identify potential biological biomarkers for ICI treatment. The results demonstrated that PSMB8/PSMB9/PSMB10/PSME1/PSME2 could be a biotarget for clinical response in multiple cohorts receiving PD-1/PDL1 and CTLA-4 therapy, and these cohorts from various centres provide us with an exciting foundation. After that, we calculated the expression levels of PSMB8/PSMB9/PSMB10/PSME1/PSME2/IRF1 as a unified score using the GSVA method. This score was dubbed IP-score, and the expression levels of immune checkpoints in different IP-score groups in urinary solid tumours were analysed. It was discovered that IP-score was closely associated with PDCD1, CD274, CTLA-4, and LAG3 expression levels in urinary solid tumours, indicating that IP-score was closely associated with tumour escape and immune depletion.

Using high-throughput sequencing technology and computational biological research, this paper creates biomarkers and scoring methodologies related to the clinical response to ICI treatment based on current public sequencing datasets. In this work, sequencing technology was employed to examine and analyse vast amounts of data, and the biological targets of clinical immunotherapy response were enhanced. To locate relevant antigens, enhance the reference database, and validate the conclusions of this work in animal models and clinical follow-ups, however, we will need to continue to innovate sequencing technology in the future.

## Conclusion

It is concluded that the transcriptome level and single-cell level PSMB8, PSMB9, PSMB10, PSME1, PSME2 and IRF1 are closely associated with CD8^+^ T lymphocyte levels in solid tumours. PSMB8, PSMB9, PSMB10, PSME1, PSME2, and IRF1 can be used as prognostic protective factors for various solid tumours and improve the overall survival of patients. In addition, PSMB8, PSMB9, PSMB10, PSME1, PSME2, and IRF1 can be used as biological markers for ICI treatment and to predict clinical response rates after receiving treatment. Finally, we scored the above six biomarkers with GSVA and constructed a scoring method for predicting immune checkpoint inhibitor therapy named IP-score.

## Data availability statement

The original data of the samples in this study can be obtained from the open source databases TCGA and GEO. The original immune therapy data could be downloaded from Immune Checkpoint Blockade therapy Atlas (ICBatlas).

## Ethics statement

This study has been conducted in accordance with ethical standards and the national and international guidelines. This study was approved by the Research Ethics Committee and all patients signed the written informed consent in china medical university.

## Author contributions

YW, KY, YG, YL, HS, and HL analysed the data and wrote the article. YW, KY, and HL designed and modified the article. YW, KY, and HL designed and modified the article. All authors contributed to the article and approved the submitted version.
