# Supplementary figures and images for: IP-score correlated to endogenous tumour antigen peptide processing: A candidate clinical response score algorithm of immune checkpoint inhibitors therapy in multiple cohorts
Source: Front Immunol. 2023 Jan 9;13:1085491. doi: 10.3389/fimmu.2022.1085491 (PMC9868931; doi:10.3389/fimmu.2022.1085491)

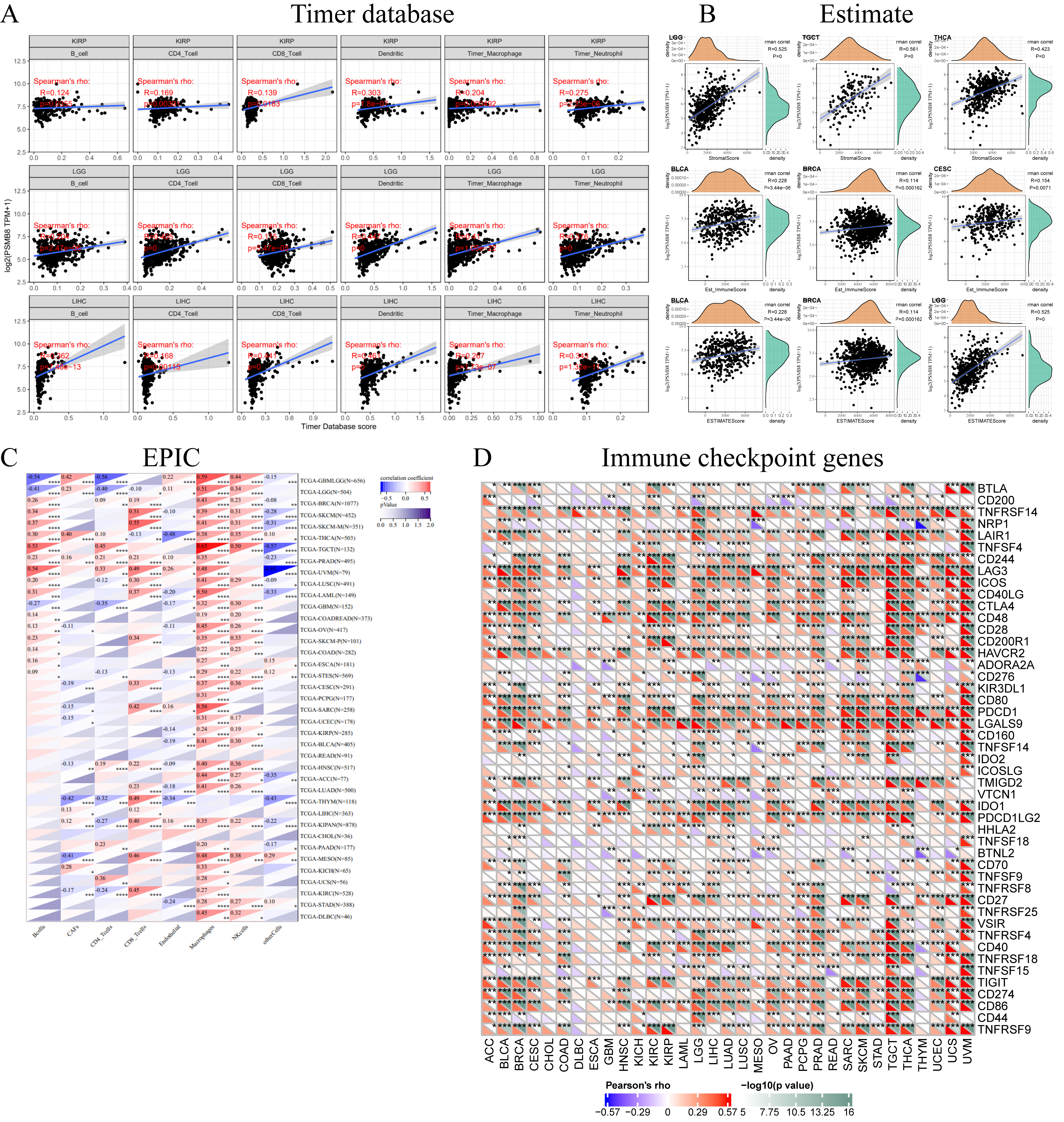

Supplement: Supplementary file 1 [file Image_1.tif]

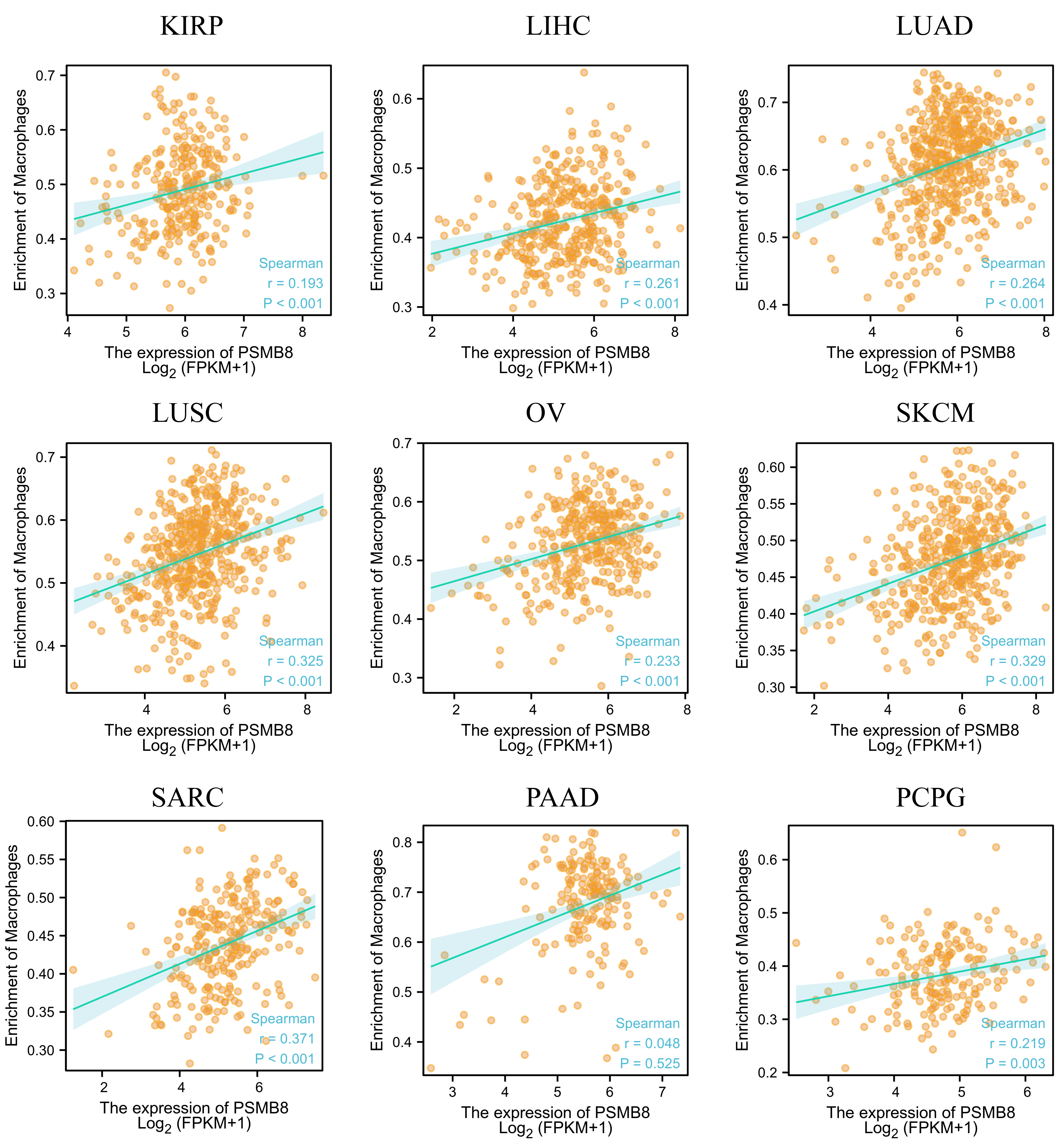

Supplement: Supplementary file 4 [file Image_4.tif]
