# Supplementary material for: IP-score correlated to endogenous tumour antigen peptide processing: A candidate clinical response score algorithm of immune checkpoint inhibitors therapy in multiple cohorts
Source: Front Immunol. 2023 Jan 9;13:1085491. doi: 10.3389/fimmu.2022.1085491 (PMC9868931; doi:10.3389/fimmu.2022.1085491)
Supplement: Supplementary file 5 [file Table_1.docx]

| **SRA** | **GEO** | **Organization** | **Contributors** | **PMID** |
| --- | --- | --- | --- | --- |
| [-](https://www.ncbi.nlm.nih.gov/sra?term= -" \t "http://bioinfo.life.hust.edu.cn/ICBatlas" \l "!/_blank) | [GSE99070](https://www.ncbi.nlm.nih.gov/geo/query/acc.cgi?acc=GSE99070) | "Submission date,May 18,2017,Last update date,Jul 25,2021,Contact name,Hyun-Sung Lee,E-mail(s),hyun-sung.lee@bcm.edu,Phone,7137988010,Organization name,BAYLOR COLLEGE OF MEDICINE,Department,Surgical Research,Lab,ABBR R651H,Street address,One Baylor Plaza,City,HOUSTON,State/province,TX,ZIP/Postal code,77030,Country,USA" | "Lee H, Jang H, Burt B" | [29618661](https://pubmed.ncbi.nlm.nih.gov/29618661) |
| [SRP094781](https://www.ncbi.nlm.nih.gov/sra?term= SRP094781" \t "http://bioinfo.life.hust.edu.cn/ICBatlas" \l "!/_blank) | [GSE91061](https://www.ncbi.nlm.nih.gov/geo/query/acc.cgi?acc=GSE91061) | "Submission date,Dec 08,2016,Last update date,May 15,2019,Contact name,Vladimir Makarov,E-mail(s),makarov@ccf.org,Organization name,Cleveland Clinic,Department,LRI,Lab,CITI,Street address,9500 Euclid Ave,City,Cleveland,State/province,OH,ZIP/Postal code,44195,Country,USA" | "Riaz N, Have JJ, Makarov V, Desrichard A, Chan TA" | [29033130](https://pubmed.ncbi.nlm.nih.gov/29033130) |
| [SRP070710](https://www.ncbi.nlm.nih.gov/sra?term= SRP070710" \t "http://bioinfo.life.hust.edu.cn/ICBatlas" \l "!/_blank) | [GSE78220](https://www.ncbi.nlm.nih.gov/geo/query/acc.cgi?acc=GSE78220) | "Submission date,Feb 23,2016,Last update date,May 15,2019,Contact name,Willy Hugo,E-mail(s),hwilly@mednet.ucla.edu,Organization name,UCLA,Department,Medicine,,Street address,UCLA Division of Dermatology 52-121 CHS, 10833 Le Conte Ave,City,Los Angeles,State/province,California,ZIP/Postal code,90066,Country,USA" | "Hugo W, Zaretsky JM, Sun L, Song C, Homet-Moreno B, Hu-Lieskovan S, Berent-Maoz B, Pang J, Chmielowski B, Cherry G, Seja E, Lomeli S, Kong X, Kelley MC, Sosman JA, Johnson DB, Ribas A, Lo RS" | [26997480](https://pubmed.ncbi.nlm.nih.gov/26997480) |
| [-](https://www.ncbi.nlm.nih.gov/sra?term= -" \t "http://bioinfo.life.hust.edu.cn/ICBatlas" \l "!/_blank) | [GSE67501](https://www.ncbi.nlm.nih.gov/geo/query/acc.cgi?acc=GSE67501) | "Submission date,Apr 01,2015,Last update date,Dec 22,2017,Contact name,Alan E. Berger,E-mail(s),alanberger@alum.mit.edu,Phone,301-938-3565,Organization name,Johns Hopkins University,Department,School of Medicine,Lab,Division of Allergy and Clinical Immunology,Street address,5501 Hopkins Bayview Circle Room 3B.74C,City,Baltimore,State/province,MD,ZIP/Postal code,21224,Country,USA" | "Ascierto ML, McMiller TL, Berger AE, Danilova L, Anders RA, Netto GJ, Xu H, Pritchard TS, Fan J, Cheadle C, Cope L, Drake CG, Pardoll DM, Taube JM, Topalian SL" | [27491898](https://pubmed.ncbi.nlm.nih.gov/27491898) |
| [-](https://www.ncbi.nlm.nih.gov/sra?term= -" \t "http://bioinfo.life.hust.edu.cn/ICBatlas" \l "!/_blank) | [GSE176307](https://www.ncbi.nlm.nih.gov/geo/query/acc.cgi?acc=GSE176307) | "Submission date,Jun 07,2021,Last update date,Sep 08,2021,Contact name,Kim Lab UNC,Phone,9199669729,Organization name,UNC-CH LCCC,Lab,William Kim,Street address,450 West Drive, Room 21-209,City,Chapel Hill,State/province,NC,ZIP/Postal code,27599-7295,Country,USA" | "Kim WY, Rose TL, Weir WH" | [34294892](https://pubmed.ncbi.nlm.nih.gov/34294892) |
| [SRP302761](https://www.ncbi.nlm.nih.gov/sra?term= SRP302761" \t "http://bioinfo.life.hust.edu.cn/ICBatlas" \l "!/_blank) | [GSE165278](https://www.ncbi.nlm.nih.gov/geo/query/acc.cgi?acc=GSE165278) | "Submission date,Jan 21,2021,Last update date,Jun 08,2021,Contact name,Bic MSKCC,E-mail(s),bic-geo-submission@cbio.mskcc.org,Organization name,Memorial SLoan-Kettering Cancer Center,Street address,1275 York Ave.,City,New York,State/province,NY,ZIP/Postal code,10021,Country,USA" | "Zappasodi R, Serganova I, Cohen I, Maeda M, Shindo M, Senbabaoglu Y, Watson MJ, Leftin A, Maniyar R, Verma S, Lubin M, Ko M, Mane MM, Zhong H, Liu C, Ghosh A, Abu-Akeel M, Ackerstaff E, Koutcher JA, Ho P, Delgoffe GM, Blasberg R, Wolchok JD, Merghoub T" | [33588426](https://pubmed.ncbi.nlm.nih.gov/33588426) |
| [SRP250849](https://www.ncbi.nlm.nih.gov/sra?term= SRP250849" \t "http://bioinfo.life.hust.edu.cn/ICBatlas" \l "!/_blank) | [GSE145996](https://www.ncbi.nlm.nih.gov/geo/query/acc.cgi?acc=GSE145996) | "Submission date,Feb 26,2020,Last update date,Nov 06,2020,Contact name,Jihye Kim,E-mail(s),Jihye.Kim@CUanschutz.edu,Phone,303-724-8229,Organization name,UCDenver,Department,Medicine,,Street address,12801 E. 17th Ave.,City,Aurora,State/province,CO,ZIP/Postal code,80016,Country,USA" | "Amato CM, Wells K, Hintzsche JD, Applegate A, Gordon N, Vorwald VM, Tobin RP, Nassar K, Kim J, Shellman Y, Medina T, Rioth M, Lewis K, McCarter MD, Tan A, Robinson WA" | [32708981](https://pubmed.ncbi.nlm.nih.gov/32708981) |
| [-](https://www.ncbi.nlm.nih.gov/sra?term= -" \t "http://bioinfo.life.hust.edu.cn/ICBatlas" \l "!/_blank) | [GSE140901](https://www.ncbi.nlm.nih.gov/geo/query/acc.cgi?acc=GSE140901) | "Submission date,Nov 24,2019,Last update date,Aug 24,2021,Contact name,Chia-Lang Hsu,E-mail(s),chialanghsu@ntuh.gov.tw,Organization name,National Taiwan University Hospita,Department,Department of Medical Research,,Street address,No. 7, Zhongshan S. Rd.,City,Taipei,,ZIP/Postal code,100,Country,Taiwan" | "Hsu C, Ou DL, Hsu CL" | [34414122](https://pubmed.ncbi.nlm.nih.gov/34414122) |
| [-](https://www.ncbi.nlm.nih.gov/sra?term= -" \t "http://bioinfo.life.hust.edu.cn/ICBatlas" \l "!/_blank) | [GSE136961](https://www.ncbi.nlm.nih.gov/geo/query/acc.cgi?acc=GSE136961) | "Submission date,Sep 05,2019,Last update date,Apr 16,2020,Contact name,Sohyun Hwang,E-mail(s),blissfulwin@cha.ac.kr,Organization name,Cha University,Department,Pathology,Street address,59, Yatap-ro, Bundang-gu,City,Sengnam,ZIP/Postal code,13496,Country,South Korea" | "Hwang S, An H" | [31959763](https://pubmed.ncbi.nlm.nih.gov/31959763) |
| [SRP217040](https://www.ncbi.nlm.nih.gov/sra?term= SRP217040" \t "http://bioinfo.life.hust.edu.cn/ICBatlas" \l "!/_blank) | [GSE135222](https://www.ncbi.nlm.nih.gov/geo/query/acc.cgi?acc=GSE135222) | "Submission date,Aug 01,2019,Last update date,Aug 21,2020,Contact name,Jeongyeon Kim,E-mail(s),kjy6690@gmail.com,Organization name,Korea Advanced Institute of Science and Technology,Street address,291 Daehakro,City,Daejeon,ZIP/Postal code,34141,Country,South Korea" | Kim H | [31537801](https://pubmed.ncbi.nlm.nih.gov/31537801) |
| [SRP183455](https://www.ncbi.nlm.nih.gov/sra?term= SRP183455" \t "http://bioinfo.life.hust.edu.cn/ICBatlas" \l "!/_blank) | [GSE126044](https://www.ncbi.nlm.nih.gov/geo/query/acc.cgi?acc=GSE126044) | "Submission date,Feb 04,2019,Last update date,Sep 04,2020,Contact name,JAE WON CHO,Organization name,Yonsei University,Department,Biotechnology,Lab,Network Biomedicine Laboratory,Street address,50 Yonsei-ro, Seodaemun-gu,City,Seoul,,ZIP/Postal code,3722,Country,South Korea" | "Cho J, Hong MH, Ha S, Kim Y, Cho BC, Lee I, Kim HR" | [32879421](https://pubmed.ncbi.nlm.nih.gov/32879421) |
| [-](https://www.ncbi.nlm.nih.gov/sra?term= -" \t "http://bioinfo.life.hust.edu.cn/ICBatlas" \l "!/_blank) | [GSE122220](https://www.ncbi.nlm.nih.gov/geo/query/acc.cgi?acc=GSE122220) | "Submission date,Nov 06,2018,Last update date,Nov 02,2020,Contact name,Zhiqiang Wang,E-mail(s),Zhiqiang Wang,Phone,713-745-8798,Organization name,University of Texas MD Anderson Cancer Center,Department,Lymphoma and Myeloma,Lab,Richard Eric Davis,Street address,7455 Fannin St.,City,Houston,State/province,Texas,ZIP/Postal code,77054,Country,USA" | "Jaiswal A, Wang Z, Davis RE, Wargo J, Curran M" | [32917656](https://pubmed.ncbi.nlm.nih.gov/32917656) |
| [SRP150548](https://www.ncbi.nlm.nih.gov/sra?term= SRP150548" \t "http://bioinfo.life.hust.edu.cn/ICBatlas" \l "!/_blank) | [GSE115821](https://www.ncbi.nlm.nih.gov/geo/query/acc.cgi?acc=GSE115821) | "Submission date,Jun 14,2018,Last update date,Mar 27,2019,Contact name,Tian Tian,E-mail(s),tt72@njit.edu,,Organization name,Children's Hospital of Philadelphia,Department,Center of Applied Genomics,Street address,3615 Civic Center Blvd,City,Philadelphia,State/province,PA,ZIP/Postal code,19104,Country,USA" | "Gao Z, Tian T" | [30127394](https://pubmed.ncbi.nlm.nih.gov/30127394) |
| [-](https://www.ncbi.nlm.nih.gov/sra?term= -" \t "http://bioinfo.life.hust.edu.cn/ICBatlas" \l "!/_blank) | [GSE111636](https://www.ncbi.nlm.nih.gov/geo/query/acc.cgi?acc=GSE111636) | "Submission date,Mar 09,2018,Last update date,Mar 11,2019,Contact name,Monica Martinez-Fernandez,E-mail(s),monicamartinezfernandez@gmail.com,Organization name,CIEMAT,Street address,Avda Complutense 40,City,Madrid,,ZIP/Postal code,28040,Country,Spain" | "Homet Moreno B, Due as M, Monica Martinez-Fernandez M, Paramio JM" | [unavailable](https://pubmed.ncbi.nlm.nih.gov/unavailable) |
| [-](https://www.ncbi.nlm.nih.gov/sra?term= -" \t "http://bioinfo.life.hust.edu.cn/ICBatlas" \l "!/_blank) | [-](https://www.ncbi.nlm.nih.gov/geo/query/acc.cgi?acc=-) | TCGA | TCGA | [unavailable](https://pubmed.ncbi.nlm.nih.gov/unavailable) |
| [ERP105482](https://www.ncbi.nlm.nih.gov/sra?term= ERP105482" \t "http://bioinfo.life.hust.edu.cn/ICBatlas" \l "!/_blank) | [-](https://www.ncbi.nlm.nih.gov/geo/query/acc.cgi?acc=-) | "Registration date: 14-Feb-2019,Melanoma Institute Australia" | "Tuba N Gide, Camelia Quek, Alexander M Menzies, Annie T Tasker, Ping Shang, Jeff Holst, Jason Madore, Su Yin Lim, Rebecca Velickovic, Matthew Wongchenko, Yibing Yan, Serigne Lo, Matteo S Carlino, Alexander Guminski, Robyn P M Saw, Angel Pang, Helen M McGuire, Umaimainthan Palendira, John F Thompson, Helen Rizos, Ines Pires da Silva, Marcel Batten, Richard A Scolyer, Georgina V Long, James S Wilmott" | [30753825](https://pubmed.ncbi.nlm.nih.gov/30753825) |
| [SRP011540](https://www.ncbi.nlm.nih.gov/sra?term= SRP011540" \t "http://bioinfo.life.hust.edu.cn/ICBatlas" \l "!/_blank) | [-](https://www.ncbi.nlm.nih.gov/geo/query/acc.cgi?acc=-) | Registration date: 3-Feb-2012:The Broad Institute (BI) | "David Liu, Bastian Schilling, Derek Liu, Antje Sucker, Elisabeth Livingstone, Livnat Jerby-Arnon, Lisa Zimmer, Ralf Gutzmer, Imke Satzger, Carmen Loquai, Stephan Grabbe, Natalie Vokes, Claire A. Margolis, Jake Conway, Meng Xiao He, Haitham Elmarakeby, Felix Dietlein, Diana Miao, Adam Tracy, Helen Gogas, Simone M. Goldinger, Jochen Utikal, Christian U. Blank, Ricarda Rauschenberg, Dagmar von Bubnoff, Angela Krackhardt, Benjamin Weide, Sebastian Haferkamp, Felix Kiecker, Ben Izar, Levi Garraway, Aviv Regev, Keith Flaherty, Annette Paschen, Eliezer M. Van Allen, Dirk Schadendorf" | [31792460](https://pubmed.ncbi.nlm.nih.gov/31792460) |
| [SRP011540](https://www.ncbi.nlm.nih.gov/sra?term= SRP011540" \t "http://bioinfo.life.hust.edu.cn/ICBatlas" \l "!/_blank) | [-](https://www.ncbi.nlm.nih.gov/geo/query/acc.cgi?acc=-) | Registration date: 3-Feb-2012:The Broad Institute (BI) | "Eliezer M. Van Allen, Diana Miao, Bastian Schilling, Sachet A. Shukla, Christian Blank, Lisa Zimmer, Antje Sucker, Uwe Hillen, Marnix H. Geukes Foppen, Simone M. Goldinger, Jochen Utikal, Jessica C. Hassel, Benjamin Weide, Katharina C. Kaehler, Carmen Loquai, Peter Mohr,14 Ralf Gutzmer, Reinhard Dummer, Stacey Gabriel, Catherine J. Wu, Dirk Schadendorf, Levi A. Garraway" | [26359337](https://pubmed.ncbi.nlm.nih.gov/26359337) |
| [SRP230414](https://www.ncbi.nlm.nih.gov/sra?term= SRP230414" \t "http://bioinfo.life.hust.edu.cn/ICBatlas" \l "!/_blank) | [-](https://www.ncbi.nlm.nih.gov/geo/query/acc.cgi?acc=-) | "Registration date: 17-Oct-2019, University of California, Los Angeles" | "Gabriel Abril-Rodriguez, Davis Y. Torrejon, Wei Liu, Jesse M. Zaretsky, Theodore S. Nowicki, Jennifer Tsoi, Cristina Puig-Saus, Ignacio Baselga-Carretero, Egmidio Medina, Michael J. Quist, Alejandro J. Garcia, William Senapedis, Erkan Baloglu, Anusha Kalbasi, Gardenia Cheung-Lau , Beata Berent-Maoz , Bego a Comin-Anduix, Siwen Hu-Lieskovan, Cun-Yu Wang, Catherine S. Grasso, Antoni Ribas" | [unavailable](https://pubmed.ncbi.nlm.nih.gov/unavailable) |
| [ERP107734](https://www.ncbi.nlm.nih.gov/sra?term= ERP107734" \t "http://bioinfo.life.hust.edu.cn/ICBatlas" \l "!/_blank) | [-](https://www.ncbi.nlm.nih.gov/geo/query/acc.cgi?acc=-) | "Registration date: 9-Apr-2018,Samsung Medical Center, Sungkyunkwan University School of Medicine, Seoul, Korea" | "Seung Tae Kim, Razvan Cristescu, Adam J. Bass, Kyoung-Mee Kim, Justin I. Odegaard, Kyung Kim , Xiao Qiao Liu, Xinwei Sher, Hun Jung, Mijin Lee, Sujin Lee, Se Hoon Park , Joon Oh Park , Young Suk Park, Ho Yeong Lim, Hyuk Lee, Mingew Choi, AmirAli Talasaz, Peter Soonmo Kang, Jonathan Cheng, Andrey Loboda, Jeeyun Lee, Won Ki Kang" | [30013197](https://pubmed.ncbi.nlm.nih.gov/30013197) |
| [SRP128156](https://www.ncbi.nlm.nih.gov/sra?term= SRP128156" \t "http://bioinfo.life.hust.edu.cn/ICBatlas" \l "!/_blank) | [-](https://www.ncbi.nlm.nih.gov/geo/query/acc.cgi?acc=-) | "Registration date: 2-Dec-2017,The Broad Institute (BI)" | "Diana Miao, Claire A. Margolis, Wenhua Gao, Martin H. Voss, Wei Li, Dylan J. Martini, Craig Norton, Stephanie M. Wankowicz, Dana Cullen, Christine Horak, Megan Wind-Rotolo, Adam Tracy, Marios Giannakis, Frank Stephen Hodi, Charles G. Drake, Mark W. Ball, Mohamad E. Allaf, Alexandra Snyder, Matthew D. Hellmann, Thai Ho, Robert J. Motzer, Sabina Signoretti, William G. Kaelin Jr. Toni K. Choueiri, Eliezer M. Van Allen" | [29301960](https://pubmed.ncbi.nlm.nih.gov/29301960) |
| [-](https://www.ncbi.nlm.nih.gov/sra?term= -" \t "http://bioinfo.life.hust.edu.cn/ICBatlas" \l "!/_blank) | [-](https://www.ncbi.nlm.nih.gov/geo/query/acc.cgi?acc=-) | "Department of Medicine, Vanderbilt University Medical Center" | "Eileen Shiuan , Anupama Reddy, Stephanie O. Dudzinski, Aaron R. Lim, Ayaka Sugiura, Rachel Hongo, Kirsten Young, Xian-De Liu, Christof C. Smith, Kimberly B. Dahlman, Renee McAlister, Beiru Chen, Kristen Ruma, Nathan Roscoe, Jehovana Bender, Joolz Ward, Ju Young Kim, Christine Vaupel, Jennifer Bordeaux, Shridar Ganesan, Tina M. Mayer, Gregory M. Riedlinger, Benjamin G. Vincent, Nancy B. Davis, Scott M. Haake, Jeffrey C. Rathmell, Eric Jonasch, Brian I. Rini, W. Kimryn Rathmell, Kathryn E. Beckermann" | [33806963](https://pubmed.ncbi.nlm.nih.gov/33806963) |
| [-](https://www.ncbi.nlm.nih.gov/sra?term= -" \t "http://bioinfo.life.hust.edu.cn/ICBatlas" \l "!/_blank) | [-](https://www.ncbi.nlm.nih.gov/geo/query/acc.cgi?acc=-) | "Department of Medical Oncology, Dana-Farber Cancer Institute" | "David A. Braun, Yue Hou, Ziad Bakouny, Miriam Ficial, Juliet Forman, Petra Ross-Macdonald, Ashton C. Berger, Opeyemi A. Jegede, Liudmilla Elagina, John Steinharter, Maxine Sun, Megan Wind-Rotolo, Jean-Christophe Pignon, Andrew D. Cherniack, Lee Lichtenstein, Donna Neuberg, Paul Catalano, Gordon J. Freeman, Arlene H. Sharpe, David F. McDermott, Eliezer M. Van Allen, Sabina Signoretti, Catherine J. Wu, Sachet A. Shukla, Toni K. Choueiri" | [32472114](https://pubmed.ncbi.nlm.nih.gov/32472114) |
| [-](https://www.ncbi.nlm.nih.gov/sra?term= -" \t "http://bioinfo.life.hust.edu.cn/ICBatlas" \l "!/_blank) | [-](https://www.ncbi.nlm.nih.gov/geo/query/acc.cgi?acc=-) | Barts Experimental Cancer Medicine Centre | "Sanjeev Mariathasan, Shannon J. Turley, Dorothee Nickles, Alessandra Castiglioni , Kobe Yuen , Yulei Wang , Edward E. Kadel III , Hartmut Koeppen , Jillian L. Astarita , Rafael Cubas , Suchit Jhunjhunwala , Romain Banchereau , Yagai Yang , Yinghui Guan , Cecile Chalouni , James Ziai , Yasin enbabao lu , Stephen Santoro , Daniel Sheinson , Jeffrey Hung , Jennifer M. Giltnane , Andrew A. Pierce , Kathryn Mesh, Steve Lianoglou , Johannes Riegler , Richard A. D. Carano , Pontus Eriksson , Mattias Hoglund , Loan Somarriba, Daniel L. Halligan, Michiel S. van der Heijden, Yohann Loriot, Jonathan E. Rosenberg, Lawrence Fong , Ira Mellman , Daniel S. Chen , Marjorie Green , Christina Derleth , Gregg D.Fine , Priti S. Hegde , Richard Bourgon, Thomas Powles" | [29443960](https://pubmed.ncbi.nlm.nih.gov/29443960) |
| [SRP155030](https://www.ncbi.nlm.nih.gov/sra?term= SRP155030" \t "http://bioinfo.life.hust.edu.cn/ICBatlas" \l "!/_blank) | [-](https://www.ncbi.nlm.nih.gov/geo/query/acc.cgi?acc=-) | "Registration date: 24-Jul-2018,Department of Systems Biology, Columbia University" | "Junfei Zhao, Andrew X. Chen, Robyn D. Gartrell , Andrew M. Silverman, Luis Aparicio, Tim Chu, Darius Bordbar , David Shan , Jorge Samanamud, Aayushi Mahajan, Ioan Filip , Rose Orenbuch , Morgan Goetz , Jonathan T. Yamaguchi, Michael Cloney, Craig Horbinski, Rimas V. Lukas, Jeffrey Raizer, Ali I. Rae, Jinzhou Yuan, Peter Canoll, Jeffrey N. Bruce, Yvonne M. Saenger, Peter Sims , Fabio M. Iwamoto, Adam M. Sonabend, Raul Rabadan" | [30742119](https://pubmed.ncbi.nlm.nih.gov/30742119) |
| [-](https://www.ncbi.nlm.nih.gov/sra?term= -" \t "http://bioinfo.life.hust.edu.cn/ICBatlas" \l "!/_blank) | [GSE93157](https://www.ncbi.nlm.nih.gov/geo/query/acc.cgi?acc=GSE93157) | "Submission date,Jan 04,2017,Last update date,Aug 15,2017,Contact name,Tomá Pascual,E-mail(s),topascual@clinic.cat,Phone,34646799556,Organization name,Hospital Clinic de Barcelona,Department,Medical Oncology,Lab,IDIBAPs,Street address,Villarroel 170,City,Barcelona,,ZIP/Postal code,8080,Country,Spain" | Prat A | [28487385](https://pubmed.ncbi.nlm.nih.gov/28487385) |
